# Introducing the PLOS collection on rare cancer

**DOI:** 10.1371/journal.pone.0308087

**Published:** 2024-07-31

**Authors:** Mitesh J. Borad

**Affiliations:** 1 Division of Hematology/Oncology, Mayo Clinic, Scottsdale, Arizona, United States of America; 2 Mayo Clinic Comprehensive Cancer Center, Phoenix, Arizona, United States of America; 3 Center for Individualized Medicine, Rochester, Minnesota, United States of America; PLOS: Public Library of Science, UNITED KINGDOM OF GREAT BRITAIN AND NORTHERN IRELAND

## Abstract

Rare cancers are defined by low incidence rates, and may lack evidence that supports uniform standards of care and relevant clinical guidelines. Rare cancers may represent up to 24% of all cancers, yet remain understudied and underappreciated in terms of their clinical and ultimately societal impact. The PLOS Rare Cancer Collection brings together a broad range of research endeavors that are being undertaken in rare cancers research ranging from basic biological evaluations to therapeutic drug development. This Overview presents a brief background to the Collection and highlights the contributions of included articles.

## Introduction

Rare cancers are defined by low incidence rates, are often understudied and thereby may lack evidence that supports uniform standards of care and relevant clinical guidelines. This can often present challenges spanning diagnosis, consensus on best treatment approaches and research funding. While there is no universally consistent definition of rare cancers, a number of agencies and institutions have made efforts to delineate numerical benchmarks towards this end. The United States FDA Orphan drug act defines rare diseases as those that affect less than 200,000 people in the United States [1]. The U.S. National Institutes of Health (NIH) defines rare cancers as those that affect fewer than 40,000 individuals [2]. In Europe the definition of rare cancers is those that have an incidence of less than 6 per 100,000 per year [3]. Beyond these stipulations, genomic contexts may confer certain groups as rare cancers even within common cancers (e.g. ROS1 fusions in lung cancer or NTRK fusions in colon cancer) or in an atypical demographic context (e.g. male breast cancers). In composite, rare cancers may represent up to 24% of all cancers [4], which lends itself to the paradox where collectively these cancers are one of the largest groups, yet remain understudied and underappreciated in terms of their clinical and ultimately societal impact. In the European Union (EU), the Joint Action on Rare Cancers (JARC) project has developed ten important recommendations which encompass broad themes such as need for improved networking, education, augmentation of research activities, engagement of patient communities and affecting governmental policies to bring about change [5].

In light of these considerations, the PLOS Rare Cancers Collection is quite timely. At the time of authoring of this Overview, the Collection includes 24 articles. These articles encompass three broad categories: (1) Outcomes research in rare cancers and rare cancer databases and registries, (2) Precision medicine, genomics, microbiome and immunological biomarkers in rare cancers and (3) Pre-clinical models, innovative diagnostics approaches and novel therapeutics for rare cancers. A selection of articles from the Collection are discussed in this Overview.

## Outcomes research in rare cancers and rare cancer databases and registries

A number of publications in this collection focus on outcomes in rare cancers, which are typically difficult to ascertain. The authors for these efforts should be commended for taking the initiative to shed light on these rare populations through the development of registries, conduct of prospective and retrospective studies and often pooling together data given the rare nature of the cancers under evaluation.

In a multi-center study Zhang and colleagues analyzed the prognostic factors impacting clear cell sarcoma of the kidney, which is a rare tumor in the pediatric population [6]. 54 patients were evaluated for demographics, outcomes and treatment measures across three hospitals in China over an approximately eighteen and half year period. The investigators found that tumor rupture was an independent poor prognostic factor and that carboplatin based regimens yielded more favorable outcomes. These findings pave the way for more focused efforts in prospective settings and additional cohorts.

Trama and colleagues delineate the establishment of a registry by the European Reference Network for the evaluation of outcomes in rare head and neck cancers [7]. The registry plans to assess natural history, prognosis, treatment effectiveness and quality of care.

A retrospective study was conducted by Ohmoto and colleagues evaluating long term outcomes and details of trabectedin administration in sarcoma patients [8]. The outcomes from the study provide support to consider long term administration in patients with sarcoma who can tolerate trabectedin.

Shin and colleagues evaluated 76 patients over an approximately 12 year period in patients with human papilloma virus (HPV) positive tonsillar cancer [9]. They identified positive resection margins and distant metastases as factors that yielded decreased disease specific survival. These data may be valuable for clinicians managing this rare disease in providing some guidance to patients with regards to outcomes for their illness.

In this collection, in a study examining prognostic markers in invasive papillary urothelial carcinoma, Kvikstad and colleagues found that age and multi-focality were associated with higher rates of recurrence and that mitotic activity index was associated with stage progression [10].

As reported by Furtado and colleagues, in patients with sarcoma, the investigators evaluated markerless motion capture (MMC) and video evaluations to ascertain functional outcomes in these patients [11]. The findings from the study provide the foundation development of a personalized exercise program and physiotherapy tool for patients.

A nested-control study conducted in women in Denmark by Pourhadi and colleagues showed that continuous but not intermittent estrogen-progestin post-menopausal therapy was associated with increased incidence of meningiomas [12]. These findings are interesting observations that support ongoing research for impact of hormones in the pathogenesis of meningiomas, which are rare tumors of the brain.

## Precision medicine, genomics, microbiome and immunological biomarkers in rare cancers

With the advent of next generation sequencing and increased immunological insights into the pathogenesis of cancer, oncology has seen an explosion in publications that depict efforts in the areas of precision medicine, genomics, microbiome research and immunological biomarkers. The application of these approaches to rare cancers in the scholarly works in this collection cover a diverse array of cancers and come from investigators from all over the globe, highlighting the broad interest in the study of rare cancers.

Choledochal cysts are an established risk factor for rare cancers of the bile ducts. In a study by Ye and colleagues characterizing gene expression profiles of samples from choledochal cysts, the investigators found that fibroblast growth factor 2 (FGFR2) and the transcription factor, CCAAT enhancer binding protein beta (CEBPB), were elevated [13]. These findings provide important insights into the pathogenic mechanisms that would lead to cancer development in these at risk individuals.

Poudel and colleagues evaluated the microbiota profiles in the bile of patients with biliary and pancreatic malignancies [14]. Notably, in patients with cholangiocarcinoma *Akkermansia* and *Achromobacter* genus bacteria were present more commonly compared to patients with pancreatic cancer, where *Rothia* genus bacteria were more common. The findings from this study could lead to additional studies to ascertain causal and mechanistic links and evaluations in larger cohorts.

The effects of a micro-RNA, miR-130b was evaluated by Danielson and colleagues, in preclinical models of leiomyosarcoma, a rare sarcoma of the smooth muscle [15]. The investigators found that TSC1 was a target gene for miR-130b and that it affected cell morphology and migration. These findings provide novel insights into the pathogenesis of leiomyosarcoma.

Moatamed and colleagues evaluated androgen receptor expression in endometrial cancers and found that it was near ubiquitous in endometrial endometrioid cancers (EECs) and prevalent in about two-thirds of non-endometrioid endometrial cancers (NEEC) [16].

In a study conducted by Mumba and colleagues from Zambia, the investigators evaluated the immune microenvironment of penile cancers and found that there was a strong correlation between biomarkers such as PD-1 expression and expression of other immune checkpoints such as TIM-3 and LAG3 [17]. Higher grade tumors in the study were shown to have higher infiltrates of FOXP3, CD68, CD163, LAG3, PD-1 and TIM3 positive cells. The study is quite impactful given that penile cancers, especially from African patients, are considerably understudied.

A study from da Silva and colleagues from Brazil evaluated L1CAM, CDX2, p53 and microsatellite instability in patients with uterine carcinosarcomas which are rare sarcomas of the uterus [18]. They found that CDX2 positivity and presence of metastatic disease were prognostic biomarkers for overall survival and progression-free survival in these patients. These findings provide impetus for multi-centered evaluations in this rare cancer.

Leroy and colleagues present their work on comprehensive genomic profiling in patients in a retrospective, multi-centered study in France [19]. Importantly, of the patients who underwent testing, 22.6% of patients had rare cancers.

The tumor suppressor 4E-BP1 and the cyclin dependent kinase, CDK1, were evaluated in T-cell lymphoma pre-clinical models by Sun and colleagues [20]. They showed that loss of 4E-BP1 resulted in development of T-cell lymphoma in the setting of radiation mediated injuries. These findings provide support for further evaluation of these biomarkers in this rare cancer.

Ma and colleagues identified the long-coding RNA, LINC00473, as a downstream mediator to DNAJB1-PRKACA fusions in fibrolamellar hepatocellular carcinoma, an extremely rare cancer of the liver afflicting young patients [21]. Given that the pathogenesis of fibrolamellar hepatocellular cancer is understudied, these novel findings may have important implications in understanding the pathogenesis of the disease.

## Pre-clinical models, innovative diagnostics approaches and novel therapeutics for rare cancers

While there has been a paucity of relevant disease models, diagnostic technologies and novel therapies being developed for rare cancers, in recent years that has been increasing interest in this regard. This has been furthered through policies by governments around the world providing financial incentives to product developers for rare ailments and there has also been increasing impetus from patient advocacy groups for rare cancers in this regard. Publications in the collection that highlight novel pre-clinical models, innovative diagnostics or novel therapies for rare cancers are described below.

A study was conducted by Murphy and colleagues in neuroblastoma, an extracranial pediatric solid tumor with challenging prognosis with 5 year survivals less than 20% [22]. The paucity of tumor models was addressed by the investigators by developing a MYC amplified cisplatin sensitive and resistance models with fluorescence reporter capabilities. Expression profiling of the models identified low levels of tumor necrosis factor receptor superfamily member 4 (TNFRSF4) as a putative prognostic factor linked to lower survival.

Pearson and colleagues evaluated CBL0137 which is derived from curaxin, in patient derived and murine models with JAK2 alterations for myeloproliferative neoplasms (MPNs) [23]. They showed that it was efficacious in these models including clinically relevant endpoints such as spleen size and reticulocyte count changes. While there are a number of JAK2 targeting therapies for MPNs, these findings provide support for additional approaches towards this end.

In a study conducted by Birnbacher and colleagues, grating based x-ray phase contrast tomography (GBPC-CT) was used as a novel imaging modality to discern between angiomyolipomas and oncocytomas and other more pathogenic kidney lesions such as renal cell carcinoma [24]. The study has important implications as incidental imaging findings are a common clinical scenario and given that biopsy would be most valuable only in those patients that have a high likelihood of having developed a malignancy compared to those who have benign disease.

Cutaneous T cell lymphoma (CTCL) is a rare skin cancer. In this study by Macleod and colleagues, the investigators evaluated the effects of ultraviolet A light and 8-methoxypsoralen on platelets assessed through aggregation studies and platelet activation markers and demonstrated that these treatments did not have an impact on these assessments *in vitro* [25].

Inoue and colleagues evaluated the application of endoscopic biliary ethanol ablation in porcine models with the objective to apply these findings to extrahepatic cholangiocarcinoma [26]. This work is of high interest as it demonstrated technical success of this novel approach with a goal towards translation to the clinic.

## Closing remarks

The manuscripts that constitute this collection exhibit the broad range of research endeavors that are being undertaken in rare cancers research ranging from basic biological evaluations to therapeutic drug development that leverages disease pathogenesis findings. By aggregating these scholarly endeavors as a coalition, the impact of work in this area is considerably amplified and provides encouragement for both researchers and patients and their families, who are afflicted by these ailments. There is also hope that these efforts are able to draw ongoing attention of research funding agencies and regulators who have been sympathetic to the plight and unique challenges faced by patients with rare cancers.

## References

[1] U.S. Food and Drug Administration. OCE Rare Cancers Program. 27 Jun 2024 [cited 7 Jul 2024]. Available from: https://www.fda.gov/about-fda/oncology-center-excellence/oce-rare-cancers-program

[2] National Cancer Institute. About rare cancers. 27 Feb 2019 [cited 7 Jul 2024]. Available from: https://www.cancer.gov/pediatric-adult-rare-tumor/rare-tumors/about-rare-cancers

[3] Information Network on Rare Cancers. Cancer list. [cited 7 Jul 2024]. Available from: http://rarecarenet.istitutotumori.mi.it/rarecarenet/index.php/cancerlist

[4] Gatta G, Capocaccia R, Botta L, Mallone S, De Angelis R, Ardanaz E, et al. Burden and centralised treatment in Europe of rare tumours: results of RARECAREnet—a population-based study. Lancet Oncol. 2017;18: 1022–1039. doi: 10.1016/S1470-2045(17)30445-X 28687376

[5] Joint Action on Rare Cancers. Rare cancer agenda 2030. 2020 [cited 7 Jul 2024]. Available from: https://www.esmo.org/policy/rare-cancers-working-group/rare-cancers-in-europe/rare-cancer-agenda-2030

[6] Zhang A, Yuan X, Jiang S, Xu D, Huang C, Tang J yan, et al. Outcomes of children with clear cell sarcoma of kidney following NWTS strategies in Shanghai China (2003–2021). PLoS One. 2024;7: e0306863. doi: 10.1371/journal.pone.0306863 38980838 PMC11233012

[7] Trama A, Licitra L, Cavalieri S, Bonfarnuzzo S, Baili P, Ciarfella A, et al. The observational clinical registry (cohort design) of the European Reference Network on Rare Adult Solid Cancers: The protocol for the rare head and neck cancers. PLoS One. 2023;18: e0283071. doi: 10.1371/journal.pone.0283071 36928072 PMC10019606

[8] Ohmoto A, Nakano K, Fukuda N, Wang X, Urasaki T, Hayashi N, et al. Clinical characteristics of sarcoma cases in which long-term disease control was achieved with trabectedin treatment: A retrospective study. PLoS One. 2023;18: e0280508. doi: 10.1371/journal.pone.0280508 36857355 PMC9977011

[9] Shin H Il, Cho KJ, Kim MS, Joo YH. Predictive factors of distant metastasis in surgically treated HPV-positive tonsil cancer. PLoS One. 2023;18: e0283368. doi: 10.1371/journal.pone.0283368 36943852 PMC10030005

[10] Kvikstad V, Lillesand M, Gudlaugsson E, Mangrud OM, Rewcastle E, Skaland I, et al. Proliferation and immunohistochemistry for p53, CD25 and CK20 in predicting prognosis of non-muscle invasive papillary urothelial carcinomas. PLoS One. 2024;19: e0297141. doi: 10.1371/journal.pone.0297141 38277354 PMC10817121

[11] Furtado S, Galna B, Godfrey A, Rochester L, Gerrand C. Feasibility of using low-cost markerless motion capture for assessing functional outcomes after lower extremity musculoskeletal cancer surgery. PLoS One. 2024;19: e0300351. doi: 10.1371/journal.pone.0300351 38547229 PMC10977781

[12] Pourhadi N, Meaidi A, Friis S, Torp-Pedersen C, Mørch LS. Menopausal hormone therapy and central nervous system tumors: Danish nested case-control study. PLoS Med. 2023;20: e1004321. doi: 10.1371/journal.pmed.1004321 38113227 PMC10729984

[13] Ye Y, Lui VCH, Babu RO, Wu Z, Wu W, Chung PHY, et al. Identification of cancer-related genes FGFR2 and CEBPB in choledochal cyst via RNA sequencing of patient-derived liver organoids. PLoS One. 2023;18: e0283737. doi: 10.1371/journal.pone.0283737 36996081 PMC10062558

[14] Poudel SK, Padmanabhan R, Dave H, Guinta K, Stevens T, Sanaka MR, et al. Microbiomic profiles of bile in patients with benign and malignant pancreaticobiliary disease. PLoS One. 2023;18: e0283021. doi: 10.1371/journal.pone.0283021 37071646 PMC10112786

[15] Danielson LS, Guijarro M V., Menendez S, Higgins B, Sun Q, Mittal K, et al. MiR-130b modulates the invasive, migratory, and metastatic behavior of leiomyosarcoma. PLoS One. 2023;18: e0278844. doi: 10.1371/journal.pone.0278844 36701370 PMC9879492

[16] Moatamed NA, Vahdatshariatpanahi S, Gjertson DW, Sachs CR, Kang Y, Ostrzega N, et al. Androgen receptor and its correlation with estrogen and progesterone receptors, aimed for identification of cases for future antiandrogen therapy in endometrial cancers. PLoS One. 2023;18: e0291361. doi: 10.1371/journal.pone.0291361 37725629 PMC10508627

[17] Mumba C, Muhimbe Z, Mapulanga V, Kawimbe M, Mutale K, Hamasuku A, et al. The effects of HIV and oncogenic human papillomavirus on the tumor immune microenvironment of penile squamous cell carcinoma. PLoS One. 2024;19: e0300729. doi: 10.1371/journal.pone.0300729 38691575 PMC11062539

[18] da Silva JL, de Albuquerque LZ, Rodrigues FR, Bastos NC, Small IA, Barroso EBC, et al. Exploring biomarkers and prognostic factors in uterine carcinosarcoma: An insight into L1CAM, CDX2, p53, and MSI status. PLoS One. 2023;18: e0285447. doi: 10.1371/journal.pone.0285447 37200263 PMC10194969

[19] Leroy K, Valette CA, Alexandre J, Boussemart L, Chiesa J, Deldycke C, et al. Retrospective analysis of real-world data to evaluate actionability of a comprehensive molecular profiling panel in solid tumor tissue samples (REALM study). PLoS One. 2023;18: e0291495. doi: 10.1371/journal.pone.0291495 37708140 PMC10501576

[20] Sun R, Guo S, Shuda Y, Chakka AB, Rigatti LH, Zhao G, et al. Mitotic CDK1 and 4E-BP1 I: Loss of 4E-BP1 serine 82 phosphorylation promotes proliferative polycystic disease and lymphoma in aged or sublethally irradiated mice. PLoS One. 2023;18: e0282722. doi: 10.1371/journal.pone.0282722 37145994 PMC10162543

[21] Ma RK, Tsai PY, Farghli AR, Shumway A, Kanke M, Gordan JD, et al. DNAJB1-PRKACA fusion protein-regulated LINC00473 promotes tumor growth and alters mitochondrial fitness in fibrolamellar carcinoma. PLoS Genet. 2024;20: e1011216. doi: 10.1371/journal.pgen.1011216 38512964 PMC11020935

[22] Murphy C, Devis-Jauregui L, Struck R, Boloix A, Gallagher C, Gavin C, et al. In vivo cisplatin-resistant neuroblastoma metastatic model reveals tumour necrosis factor receptor superfamily member 4 (TNFRSF4) as an independent prognostic factor of survival in neuroblastoma. PLoS One. 2024;19: e0303643. doi: 10.1371/journal.pone.0303643 38809883 PMC11135766

[23] Pearson S, Blance R, Yan F, Hsieh YC, Geary B, Amaral FMR, et al. Identification of curaxin as a potential new therapeutic for JAK2 V617F mutant patients. PLoS One. 2023;18: e0286412. doi: 10.1371/journal.pone.0286412 37253035 PMC10228771

[24] Birnbacher L, Braunagel M, Willner M, Marschner M, De Marco F, Viermetz M, et al. Quantitative differentiation of minimal-fat angiomyolipomas from renal cell carcinomas using grating-based x-ray phase-contrast computed tomography: An ex vivo study. PLoS One. 2023;18: e0279323. doi: 10.1371/journal.pone.0279323 37058505 PMC10104346

[25] Macleod H, Weiss L, Kelliher S, Kevane B, Áinle FN, Maguire PB. The effect of UVA light/8-methoxypsoralen exposure used in Extracorporeal Photopheresis treatment on platelets and extracellular vesicles. PLoS One. 2024;19: e0293687. doi: 10.1371/journal.pone.0293687 38416722 PMC10901342

[26] Inoue T, Kutsumi H, Ibusuki M, Yoneda M. Endoscopic biliary ethanol ablation using a novel multi-hole balloon catheter: In vivo feasibility study in a swine model. PLoS One. 2023;18: e0283733. doi: 10.1371/journal.pone.0283733 37000862 PMC10065430

